# Investigating the prevalence of drowning in East Azarbaijan province during the years 2018-2022 

**Published:** 2022-12

**Authors:** Sahar Barzegar, Farzad Rahmani, Babaei Javad, Mehrdad Abbasqolizad Toloui, Abazar Fathollahzadeh

**Affiliations:** ^*a*^ Pre-Hospital Emergency Department and Incident Management, Tabriz, Iran.; ^*b*^ Traffic Injury Research Center, Tabriz University of Medical Sciences,Tabriz, Iran.; ^*c*^ Department of Health Policy and Management, School of Management and Medical Informatics, Tabriz University of Medical Sciences, Tabriz, Iran.; ^*d*^ Department of Health in Disasters and Emergencies, Faculty of Health, Shahid Sadougi University of Medical Sciences, Yazd, Iran.

## Abstract

**Background:**

Drowning is one of the preventable events that lead to neurological damage and death due to hypoxia. It is the third cause of injuries leading to unintentional death in the world. According to forensic reports, in 2019 the number of drowned people was 1072, of which 170 were women (15.8%) and 902 were men (84.2%). However, in 2016, there were 1026 drowning cases. Therefore, this study aimed to investigate the prevalence of drowning in East Azarbaijan province.

**Methods:**

This descriptive, cross-sectional study was carried out in East Azarbaijan province. Data collection was done using the data of drownings recorded in the emergency incident registration system of East Azarbaijan province. The data were analyzed using SPSS 19 software.

**Results:**

Based on data analysis, the number of drowning cases between 2018 and 2021 is as follows: in 2018, 11 cases (10 dead and 1 injured), and in 2019, 7 cases (6 dead and 1 injured) were registered. Moreover, 7 cases (1 dead and 6 injured) in 2020, and 15 cases (11 dead and 4 injured) were recorded in 2021. The highest number of drownings were in garden pools, factories, and city dams, and among them, the most drowning cases were related to the age groups of 4 to 8 years, 18 to 37 years, and men.

**Conclusions:**

East Azarbaijan does not have a high death rate in the country with 0.49 per 100,000 people per year. However, drowning is a problem that mostly affects children and young people. According to the obtained results, special plans and interventions should be made in this field, and by informing young people about the dangers and causes of drowning, teaching swimming techniques, and lifeguard training, the occurrence of drowning can be minimized to a great extent.

**Keywords:**

Drowning, Outbreak, Incidents, East Azarbaijan

